# The Yin-Yang of DNA Damage Response: Roles in Tumorigenesis and Cellular Senescence

**DOI:** 10.3390/ijms14022431

**Published:** 2013-01-25

**Authors:** Xiaoman Li, Hongde Xu, Chongan Xu, Meina Lin, Xiaoyu Song, Fei Yi, Yanling Feng, Kathleen A. Coughlan, William Chi-shing Cho, Sang Soo Kim, Liu Cao

**Affiliations:** 1 Key Laboratory of Medical Cell Biology, Ministry of Education, China Medical University, Shenyang 110001, China; E-Mails: disneyer@hotmail.com (X.L.); xuhongdesy@hotmail.com (H.X.); lincmu@126.com (M.L.); xysong@mail.cmu.edu.cn (X.S.); yifei1983105@163.com (F.Y.); nakata_72@163.com (Y.F.); 2 Department of Medical Oncology, The Fourth Affiliated Hospital, China Medical University, Shenyang 110032, China; E-Mail: cmu4h-xca@126.com; 3 Section of Molecular Medicine, Department of Medicine, University of Oklahoma Health Sciences Center, Oklahoma City, OK 73104, USA; E-Mail: kathleen-coughlan@ouhsc.edu; 4 Department of Clinical Oncology, Queen Elizabeth Hospital, Hong Kong; E-Mail: williamcscho@gmail.com; 5 Radiation Medicine Branch, National Cancer Center, Goyang, Gyenggi 410-769, Korea

**Keywords:** DNA damage response, cell cycle arrest, tumorigenesis, senescence

## Abstract

Senescent cells are relatively stable, lacking proliferation capacity yet retaining metabolic activity. In contrast, cancer cells are rather invasive and devastating, with uncontrolled proliferative capacity and resistance to cell death signals. Although tumorigenesis and cellular senescence are seemingly opposite pathological events, they are actually driven by a unified mechanism: DNA damage. Integrity of the DNA damage response (DDR) network can impose a tumorigenesis barrier by navigating abnormal cells to cellular senescence. Compromise of DDR, possibly due to the inactivation of DDR components, may prevent cellular senescence but at the expense of tumor formation. Here we provide an overview of the fundamental role of DDR in tumorigenesis and cellular senescence, under the light of the Yin-Yang concept of Chinese philosophy. Emphasis is placed on discussing DDR outcome in the light of *in vivo* models. This information is critical as it can help make better decisions for clinical treatments of cancer patients.

## 1. Introduction

### 1.1. DNA Damage

The integrity and fidelity of DNA is pivotal for accurately passing genetic information from generation to generation. However, over an individual’s lifespan, DNA is constantly exposed to exogenous and endogenous insults. Exogenous sources of damage can come from harmful chemicals, ultraviolet light (UV) and ionizing radiation (IR), whereas endogenous hazards arise from reactive oxygen species (ROS) produced in normal metabolic processes, telomere shortening induced by cell division and “DNA replication stress” imposed by activation of oncogenes or inactivation of tumor suppressor genes. In response to DNA damage, organisms are capable of launching repair mechanisms, predominantly homologous recombination (HR) and non-homologous end joining (NHEJ), to counteract the potential damage. HR is mostly error-free, requiring an intact sister chromatid as a template for repair, by contrast, NHEJ is error-prone due to the lack of an intact template. The molecular mechanisms of DNA damage repair are not the primary focus of our review as this has been comprehensively reviewed by Thompson *et al.* [1].

### 1.2. DNA Damage Response (DDR)

**Figure 1:**
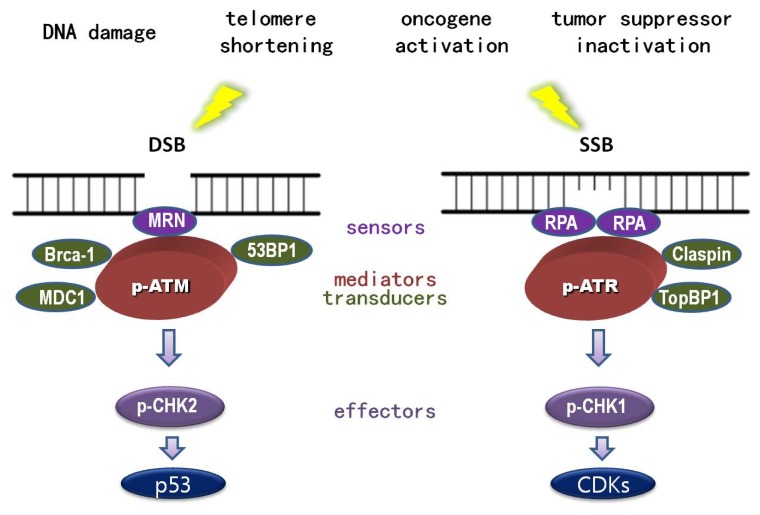
DNA damage and DNA damage response (DDR). Both external insults and internal hazards can cause DNA damage. DNA damage response is coordinated by various proteins whose functions can be categorized as DNA damage sensors, transducers, mediators, and effectors. Double strand DNA damage (DSB) can be detected by MRN complex (sensor) to recruit and activate transducer ATM (ataxia Telangiectasia mutated) to activate CHK2 (effector), with the help of DDR mediators MDC1 (mediator of DNA damage checkpoint), 53BP1 (p53-binding protein 1), and BRCA1 (breast cancer 1). In contrast, single strand DNA damage (SSB) could be detected by sensor protein, RPA (replicative protein A), to recruit and activate transducer ATR (ataxia telangiectasia- and Rad3-related), to activate CHK1 (effector), with the help of mediators TopBP1 (topoisomerase-binding protein 1) and Claspin. p53 and CDKs are the major downstream substrates in response to DSB and SSB respectively.

In addition to repair mechanisms, individuals have evolved a so called “DNA damage response”, which is responsible for invoking a myriad of cellular events in response to genotoxic stress. DNA damage response is mainly mediated by the activation of ATM (ataxia telangiectasia mutated)-CHK2 (cell cycle the checkpoint kinase 2)-p53 and ATR (ataxia telangiectasia and rad3-related)-CHK1 (cell cycle checkpoint kinase 1)-CDKs (Cyclin-dependent kinases) pathways. Once activated, these signaling cascades can trigger cell cycle arrest (so called “checkpoint”), thereby gaining time for DNA damage repair and preventing the propagation of damaged cells [2–5]. ATM and ATR belong to the same family and share some functional redundancy. However, these proteins are distinct because they respond to different aberrant DNA structures. ATM, in principle, is elicited by double-strand breaks (DSB) and recruited via interaction with DSB sensors, MRN complex (MRE11-RAD50-NBS1) [6–12]. In contrast, ATR is induced by single-strand breaks (SSB) and engaged by its partner protein, ATRIP (ATR interacting protein) through interaction with the SSB sensor RPA (replicative protein A) [13–17]. Consequently, phosphorylated ATM and ATR, acting as transducer proteins, can active the effector kinases CHK1 and CHK2, with the help of the mediator proteins; MDC1 (mediator of DNA damage checkpoint), 53BP1 (p53-binding protein 1), BRCA1 (breast cancer 1) for ATM, and TopBP1 (topoisomerase-binding protein 1) and Claspin for ATR [1,18,19] (Figure 1). Under normal circumstances, p53 is easily degraded and hence rarely detected. Upon stress, p53 is phosphorylated and stabilized following ATM and, to a less extent, ATR stimulation [20–23]. Additionally, p53 stabilization can be achieved through ARF (alternate reading frame) activation imposed by oncogene-induced replication stress [24]. The stimulation of ARF relieves the inhibitory effect of MDM2 (mouse double minute 2 homolog) on p53 [25–27]. Once p53 is stabilized and activated, it can orchestrate a range of cellular stress responses including cell cycle arrest, senescence and apoptosis. The various outcomes are determined by the intensity of stress as well as the tissue and cellular context [28,29].

## 2. Pathways of Senescence-Associated Cell Cycle Arrest

**Figure 2:**
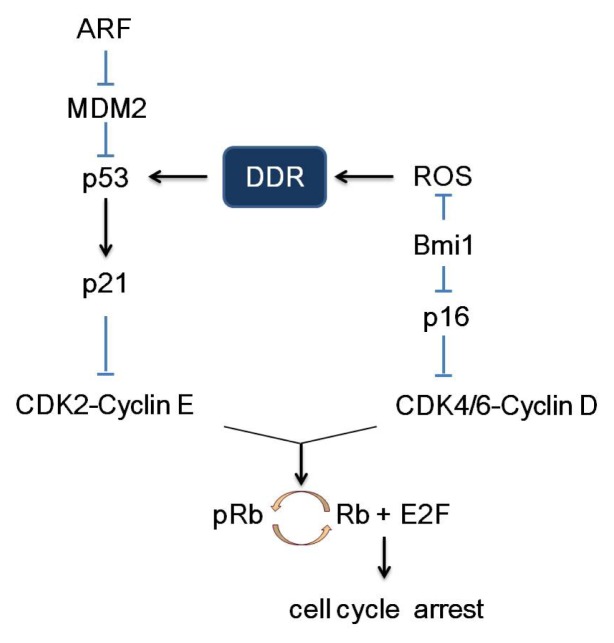
p53/p21/pRb/E2F and p16/pRb/E2F signaling pathway. The *INK4a/ARF* locus encodes both ARF (alternate reading frame) and p16 protein. ARF could stimulate p53 through inhibition and degradation of MDM2 (mouse double minute 2 homolog). Activated p53 transactivates p21, which, in turn, inhibits CDK2/cyclin E with the consequent inhibition of CDK2-dependent phosphorylation of Rb. Unphosphorylated Rb suppresses the function of the G1/S phase-promoting factor, E2F, and as a result, cells are subjected to proliferation arrest and DNA damage repair. Similarly, p16 can inhibit the CDK4-6/cyclin D complex thereby reducing Rb phosphorylation and subsequent downstream signal transduction pathways. Thus, cells will arrest in G1 phase and fail to complete the cell cycle. Bmi1, a polycomb group protein, is a transcriptional repressor of p16. Bim1 also has a potential inhibitory role on reactive oxygen species (ROS) production [33].

The cell cycle, comprised of S phase (DNA synthesis), M phase (mitosis) and two gap phases (G0 and G1), is coordinately regulated by cell cycle proteins (cyclins), cyclin-dependent kinases (CDKs) and cyclin-dependent kinase inhibitors (CDKIs). CDKIs are negative modulators of cell cycle and hence also viewed as tumor suppressor genes. CDKIs can be grouped into two categories, the KIP/CIP family (p21^Cip1^, p27^Kip1^ and p57^KipII^) and the INK4 family (p16^Ink4a^, p15^Ink4b^, p18^Ink4c^ and p19^Ink4d^). As illustrated in Figure 2, through cellular events triggered by genomic stress, stimulated p53 transactivates p21, which, in turn, inhibits CDK2/cyclin E and thereby retains Rb (Retinoblastoma) in an inactive unphosphorylated state. Unphosphorylated Rb suppresses the function of the G1/S phase-promoting, E2F, and as a result, cells are subjected to proliferation arrest and DNA damage repair [30–32]. Compelling evidence also points to a critical role of p16 as one of the central modulators of cell cycle arrest. The p16 can inhibit the CDK4-6/cyclin D complex thereby reducing Rb phosphorylation and subsequent downstream signal transduction pathways. Thus, cells will arrest in G1 phase and fail to complete the cell cycle. Since both pathways engage pRb, it is plausible to speculate proliferation arrest, in response to cellular stress, which is coordinately regulated by p53/p21/pRb/E2F and p16/pRb/E2F signal transduction pathways.

Senescence is a permanent form of cell-cycle arrest, first discovered in normal human fibroblasts by Hayflick [34]. Unlike normal cells, senescent cells are relatively stable, lacking proliferation capacity but retaining metabolic activity. These cells possess large and flattened morphology, increased intracellular particles, as well as enhanced senescence-associated β-galactosidase (SA-β-gal) activity. Under normal circumstances, as cells cycles, telomere, a special structure at the ends of chromosomes, is gradually shortened. When the length of the telomere reaches a certain limit, cell proliferation is halted and cellular senescence is elicited [35]. Such senescence is known as replicative senescence. In addition to the replicative senescence, senescence can also result from DNA damage aroused from oxidative stress or oncogene activation-induced replication stress, which is termed as premature senescence, or oncogene-induced senescence (OIS) [36–39].

Consistent with the notion that senescence is a permanent form of cell cycle arrest, factors central to checkpoint events, such as p53, p21, p16 and Rb, are also key regulators of the senescence program. In human cells, replication senescence is commonly dependent on p53/p21/pRb/E2F pathway, whereas premature senescence can be mediated through p53/p21/pRb/E2F pathway, p16/pRb/E2F pathway or both [35,40]. Mechanistically, little is known as to how a cell chooses one way over the other, however, some evidence implies that it might be associated with types of stimulus and cell context [41–44]. Given the complexity of cellular responses to various stimuli, the chances are that these pathways could be cooperative and intertwined in stress-induced senescence and associated cell cycle arrest [45,46].

## 3. DDR Is the Common Link between Tumorigenesis and Senescence

It has been known for decades that constitutive activation of oncogenes, such as Ras, is capable of driving the proliferation of malignant tumor cells [47]. Together with the observations that expression of the same oncogenes in normal cell culture leads to cell senescence rather than cell transformation [36,48,49], it raises an obvious question as to how oncogenes lead to both tumor and senescence in a similar scenario. One explanation to reconcile the paradox is that oncogene-induced DDR and resultant senescence may occur prior to tumorigenesis, imposing an intrinsic barrier to the development of the malignant tumor. In effect, a large body of emerging evidence from both bench and clinical works supports this conception [50–52]. In the early stages of tumor formation, many factors along the DDR pathway, such as ATM, CHK1, CHK 2, p53 and p16, as well as markers of DNA damage foci, such as H2AX and 53BP1, could be detected, mostly in their phosphorylated activated form [50,51,53–58]. Senescent cells and their specific markers, such as SA-β-Gal, were also present in precancerous lesions [53,59–64]. The correlation between markers of activated DDR with those of cellular senescence reinforces the crucial role of DDR signaling in oncogene-induced senescence [50,51,65]. Remarkably, markers for senescence-associated DDR were attenuated or absent in the later stage of cancer [53,59–62], leading to the speculation that senescence impinges selective pressure on hyper-proliferative tumors with mutations of checkpoint genes. Agreeably, cells escaped from OIS by depletion or inhibition of several important DNA damage signaling factors, such as 53BP1, ATM, CHK1, CHK2, and p53, predisposes to cell proliferation and oncogenic transformation [36,50,51,53,54,57,58]. Collectively, based on the results summarized above, the following concepts are emerging: (1) DNA damage is the common driving force for both tumorigenesis and cellular senescence (2) senescence-associated DDR acts as a natural barrier for tumorigenesis, and abrogation of the barrier may rescue defective cell growth and limit cell senescence in the incipient malignant form but at the expense of tumor progression [66–68]. The outcome as to whether cells predispose to tumor or senescence upon DNA damage is largely dependent on the competence of DDR signaling. To achieve the “Yin-Yang balance” of DDR, signaling is critical for preventing both tumors and senescence. The Yin and Yang of DDR in tumors and senescence could be further exemplified in the light of a number of *in vivo* mice models with genetic deletions of certain genes involved in the DDR pathways.

### 3.1. p53

Given the critical role of p53 as the “guardian of the genome”, its activity must be tightly regulated. Either too much, or too little p53 activity will have adverse effects, as demonstrated comprehensively in various mouse models using gene manipulation to alter p53 levels [69,70]. The p53 null mice are largely tumor-prone, consistent with the fact that p53 mutations are the most prevalent mutations in human cancers [71–74]. By contrast, mice with high p53 activity, such as p53^+/m^ mice, are less cancer-prone compared to the control mice, but display obvious age-related phenotypes such as tissue atrophy [69,75]. The overall high p53 activity in p53^+/m^ mice was attributed to the stabilization and enhancement of the p53 by the m allele product [69]. These findings clearly demonstrated the existence of a delicate balance between the tumor suppression and age promoting functions of p53. To optimize the outcome of p53-dependent DNA damage response and tip the Yin-Yang balance between tumor suppression and age promoting it is crucial to involve p53 in clinical applications. Cancer protection without negatively affecting aging was observed in mice containing an extra copy of p53 (super-p53 mice) [76,77]. In contrast to the p53^+/m^ mice model, the desired phenotype from super-p53 mice might be attributed to the maintenance of the normal regulation of p53 activity. Nevertheless, super-p53 mice raise great hopes of involving p53 as a potential anti-tumor treatment and further studies are needed to ascertain whether this scenario could be sustainable in humans.

### 3.2. p21 and p27

It is commonly noted that p53-induced senescence is executed, at least partly, via p21 upregulation [45]. The p21 levels are elevated in prematurely senescing fibroblasts in both humans and mice displaying premature aging syndromes, and p21^−/−^ mice exhibited deficiencies in senescence response in comparison to WT mice after UV damage [78–81]. In support of the notion that p53/p21 is the major determinant in response to telomere-dysfunction-aroused replicative senescence, p21 deficiency could reverse the short lifespan of mice with telomerase deficiency (Terc^−/−^) [82,83]. By contrast, lacking p53 increases genomic instability and cancer formation *in vivo*, thereby reducing the lifespan of mice with dysfunctional telomeres [84,85]. In the early age of p21^−/−^ mice, tumor is rarely detected and an increased tumor formation occurs at an average age of 16 months, with the most common tumor types being sarcomas and B cell lymphomas [86,87]. Mice with genetic deletion of DDB2 (damaged DNA binding protein 2), a significant player in recognizing DNA damage in NER (nucleotide excision repair), exhibited reduced senescence response and induced enhanced incidence of UV-induced skin cancers in comparison to wild-type mice [81,88–91]. A stronger inhibition of premature senescence and accelerated tumor formation in the DDB2^−/−^ p21^−/−^ mice compared to the DDB2^−/−^ single knockouts, further support the tumor-suppressive role of p21 [92]. However, taking into consideration the extremely low incidence of p21 mutations in human cancer and the less drastic tumor-prone phenotype in p21-deficient mice in comparison to mice deficient in other tumor suppressors, such as p53 or p16, the tumor suppressive role of p21 is not viewed as crucial. Recently, the tumor suppressive role of p21 was complicated by findings indicating that p21 has an inhibitory role in apoptosis [93–95]. The involvement of p21 in these pathways could confer its oncogenic activities, particularly in lymphomas [96,97].

Similar to p21, p27, another member from the KIP/CIP family, binds and inactivates CDK2-cyclin E and CDK2-cyclin A complex thereby inhibiting cell-cycle progression [98]. The p27 expression is frequently reduced in human epithelial cancers, and is correlated with tumor progression and poor survival [99–103]. Targeted disruption of p27 in the mouse model leads to multi-organ hyperplasia, loss of senescence markers and an increased tumor latency [104–108].

### 3.3. BRCA1

**Figure 3:**
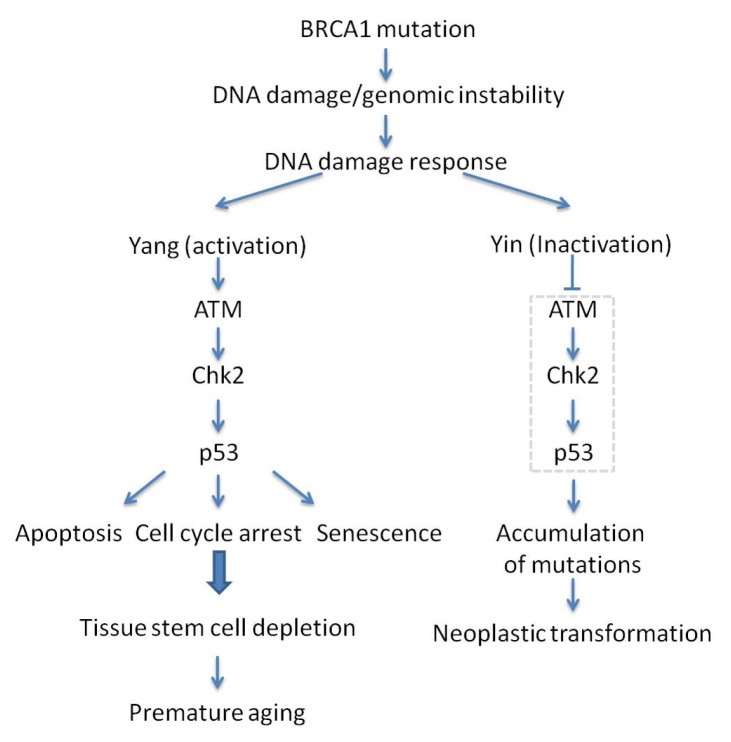
Yin and Yang of the ATM-CHK2-p53 signaling pathway upon BRCA1 mutation-associated premature aging and tumorigenesis. ATM-CHK2-p53 signaling pathway senses DNA damage/genomic instability and acts as a gatekeeper to eliminate mutations, but, as a side effect, it may also lead to premature aging.

The tumor suppressor BRCA1 is intimately associated with an increased risk of breast and ovarian cancer [109–114]. BRCA1 is a DNA damage repair protein crucial for maintaining genomic stability. The abrogation of the full-length isoform leads to genomic instability and embryonic death [114–118]. This lethal phenotype is partially due to the excessive activation of the ATM-CHK2-p53 axis which leads to accelerated aging, in response to untimely repaired chromosome breaks in the absence of BRCA1 [119,120]. In support of this notion, loss of p53 prolonged the survival of BRCA1 mutant embryos from E7.5 to E9.5 [121]. Moreover, haploid loss of p53 in BRCA1 null mice (BRCA1^Δ11/Δ11^ p53^+/−^) could completely overcome embryonic lethality, but display cancer susceptibility mostly in female mice, and premature aging, mainly in male mice [119,122]. The survival of BRCA1^Δ11/Δ11^ p53^+/−^ mice makes it a surrogate model to study the link between BRCA1 and aging. Consistent with the contribution of DDR cascade in BRCA1 deficiency, enhanced ATM and CHK2 activity was observed in BRCA1^Δ11/Δ11^ embryos. Consistently, absence of ATM, CHK2 or 53BP1 can mean escape of embryonic lethality in BRCA1 knockout mice and suppression of accelerated aging albeit at the expense of entering a tumor-prone state [120,123] (Figure 3). These observations highlight the critical role of genetic integrity, whose compromise may disturb organismal homeostasis and result in senescence and cancer.

### 3.4. p16 and Bmi1

It is evident that p16, another important component within the DDR network, is largely involved in the senescence program, acting not only as an effector but also a biomarker of senescence [124–129]. The expression of p16 is markedly elevated with age in both rodents and human tissues [128,129]. Nevertheless, mice deficient in p16 displayed an increased incidence of tumorigenesis and loss of inactivation of p16 accounts for more than 30% of human tumors [130–133]. Hence, p16 is ubiquitously linked to both tumorigenesis and senescence. Bmi1, a polycomb group protein, is a transcriptional repressor of p16. Overexpression of Bmi1 could extend the replicative life span of primary cells but promote the formation of lymphomas [42,134–137]. Conversely, mouse embryonic fibroblasts deficient in Bmi1 possess high p16 activity and undergo premature senescence, which can be relieved partially in the absence of p16 [42]. However, the median survival of Bmi1^−/−^ mice cannot be extended by p16 deficiency, but instead it could be improved by complete loss of CHK2 [33]. It is postulated that this may be due to the inhibition of ROS-induced DDR.

Collectively, p53/p21 and p16/Rb pathways are not only important for DDR signaling, but also critical in maintaining cellular and genomic homeostasis. Several lines of evidence indicate p53/p21 and p16/Rb pathways are collaborative. For instance, MEF derived from p21 and p16 double knockout mice displayed no evidence of cellular senescence to Ras-induced senescence and have a higher incidence of cancer compared to either of the single KO mice [138,139].

## 4. Will the Yin-Yang of DDR Be Beneficial for Clinical Treatment of Cancer?

The key mechanism of the most prevalent cancer therapy, radiation therapy and chemotherapy, is to damage DNA and consequently trigger DDR, tumor growth arrest, apoptosis and senescence [140–142]. The extensive DNA damage induced by these current therapies inevitably puts patients to severe side effect risk such as hair loss and bone marrow suppression [143]. Given the frequent loss of critical DDR proteins in cancer, new possibilities for tumor intervention have been postulated to re-establish the barrier or even induce tumor to senescence through exogenously introducing or molecular targeting of proteins involved in the DDR signaling. Recent studies found that chemotherapy or gene therapy, by modulating the activity of p16, p53, pRb or p21, could navigate tumor cells to senescent cells and have a substantial therapeutic effect on tumor inhibition [61,144–146]. More recently, a number of senescence-inducing small molecules entered clinical trials [147–151]. Certainly, the safety of pro-senescence therapy needs to be carefully evaluated before translating it into a clinically relevant context. Senescence is frequently accompanied with oxidative stress, altered tissue microenvironment and release of inflammatory cytokines [147,152,153]. All of these could potentially promote cancer and aging phenotypes [40,154]. Greater understanding of the molecular mechanisms involved in senescence and tumors will provide valuable new insights into how to bypass undesired side effects in senescence-inducing treatment. In the near future, it is warranted to consider the combination of pro-senescence strategies with already established treatments.

## 5. Conclusions

Organisms are natural perfectionists and dedicated hard workers. In case of any threat, a myriad of mechanisms engage in an intricate interplay to keep damage to a minimum. Networks of molecules have evolved to work coordinately to maintain the homeostasis of the body. However, imbalanced DNA damage response, upon genotoxic stress, can endanger cellular homeostasis, leading to the transition from a healthy to a disease state, including senescence and cancer. As we learned from ancient Chinese philosophy, finding the balance between “Yin and Yang” will ensure both health and longevity.
